# Errors in packaging surgical instruments based on a surgical instrument tracking system: an observational study

**DOI:** 10.1186/s12913-019-4007-3

**Published:** 2019-03-19

**Authors:** Xiaolian Zhu, Lan Yuan, Tianyi Li, Ping Cheng

**Affiliations:** Central Sterile Supply Department, The First Affiliated Hospital of Soochow University, 215008 Suzhou City, Jiangsu Province, China

**Keywords:** Central sterile supply department, Traceability information management system, Surgical instrument, Package

## Abstract

**Background:**

Surgical instrument processing is important for improving the safety of surgical care in hospitals. However, it has been rarely studied to date. Errors in surgical instrument processing may increase operative times and costs, and increase the risk of surgical infections and perioperative morbidity. We aimed to investigate the errors occurred in packaging surgical instruments.

**Methods:**

Surgical instrument tracking system in a central sterile supply department (CSSD) was used to collect the packaging data during January–August 2016 in the First Affiliated Hospital of Soochow University, Suzhou City, China.

**Results:**

Data on 33,839 surgical instrument packages were collected. A total of 398 (1.18%) errors occurred, including incomplete packages (*n* = 70), instrument missing (*n* = 77), instrument malfunction (*n* = 27), instrument in wrong specification (*n* = 175), wrong packaging tag (*n* = 8), box and cover mismatched (*n* = 14), wrong packing material (*n* = 15), indicator card missing (*n* = 6), and wrong count of instruments (n = 6). The highest error rates were observed among least experienced nurses (N1 level) and during the 16:00–20:00 time period (both *p* < 0.05). A relatively high error rate was detected in the Department of Orthopedics as well as in the Department of Gynecology and Obstetrics.

**Conclusion:**

Wrong instrument specifications were the primary packing error identified in the current study. Further effort is needed to standardize the packing procedure for instruments under the same category and more effort is required to reduce the error rate during high risk times, or in the surgery department.

## Background

Improvement in surgical quality has drawn public attention in recent years, as more studies report major complications occurred in inpatient surgical procedures that are proven to be associated with hospital death. [1, 2] Surgical quality can be influenced by various factors in clinics, such as clinicians’ skill, and the management of surgical instruments and supplies [3]. Various methods have been used to improve surgical quality in clinics [4], including surgical team checklists [5], evidence-based protocols [6], surgeon performance [7], and staff education [8]. However, as far as we know, only one study has focus on improving quality and safety in surgical sterile instrument processing [3].

Every year, typical hospitals organize a tremendous amount of instruments and therefore they could make many errors during the instruments processing, which might involve various processes, including manual cleanout for instruments, placing instruments into automated washers, manual assembly of instruments sets, instrument set packaging for sterilization, and finally the sterilization of the instrument sets. Accurate management of surgical instruments decreases the risk of operating room miscounting and malfunction. In China, most hospitals have established a central sterile supply department (CSSD) to centrally manage and track the packaging of recycled surgical instruments [9]. Recycling, cleaning, sterilization, inspection, packaging, and delivery are centrally controlled through the surgical instrument tracking systems in CSSD, which have been demonstrated to reduce the risk of errors in packaging surgical instruments, including missing and mismatched instruments [10].

Surgical instrument tracking systems have been available for use in health care facilities for the past 20 years [11]. In the past decade, there has been an evolution in tracking systems as new technology, faster information technology systems, and smart phones and tablets have been incorporated into the health care arena. Previously, these systems were simply used to manage instrument count sheets and for basic instrument traceability [11].

The introduction of two-dimensional (2D) barcodes and 2D adhesive dots has allowed sterile processing staff members to more accurately identify instruments and establish a history of each instrument’s use, which can help determine if an instrument needs to be repaired or how to better optimize set usage. In addition to the evolving unique identifier of surgical instruments, radio-frequency identification (RFID) technology is also available to health care facilities [12, 13]. RFID technology can be used to identify, track, and issue portable medical devices and help establish and clarify ownership of a device within the health care facility [13].

Surgical instrument tracking systems have been proven to save time, improve quality of care, enhance patient and staff safety, and decrease costs [14]. However, packaging errors are still reported occasionally and the reasons are unclear. Therefore, to increase the safety monitoring of surgical instrument processing, the current study aimed to discover the types of errors that occurred in surgical instrument packaging. .

## Methods

### Data collection

The surgical instrument tracking system in CSSD was routinely applied to collect the surgical instrument packaging error data during January–August 2016 in our hospital. The tracking system records data including names of staff dealing with the packing, packing time, and types of packing errors. Summary data for packing errors was automatically calculated by the tracking system. Furthermore, there is an information communication platform routinely working between CSSD and the operating room. According to hospital regulations, packaging error-related information must be shared in the information communication platform immediately after the error is identified. For example, nurses in the operating room (responsible for uploading the unique identification code of the surgical instrument package) identified errors in the information communication platform. CSSD nurses were then responsible to record that information in the tracking system.

After cleaning and sterilization, the unique identification code of each surgical instrument was scanned. The content list of the surgical instrument package was printed, followed by a check (under a magnifying lens) of the sterilization and function of the surgical instrument. All confirmed instruments were then placed in the packing basket and labelled with a chemical indicator card attached. Nurses were responsible for the entire packaging process. The head of the CSSD nurses was responsible for assigning nurses with various hierarchies to different regions in CSSD, and nurses finalized the entire packaging process independently.

### Classification of packaging errors

*Packing incomplete* includes superficial incomplete packing materials, such as damaged cotton cloth and non-woven fabrics, broken packaging tape, or lock of the containing box or cushion of the containing box are missing. *Instrument missing*: The number of instruments in the package is less than that stated on the list. Instrument malfunctions were mainly due to missing screws, instrument accessories missing, or damaged instruments. *Instrument in wrong specification* was defined when same category instruments were set with the wrong specification, or assembled incorrectly. *Wrong packaging tag* includes incomplete or disordered packaging tag. *Box and cover mismatched*: Instrument containing boxes and cover usually assigned together with different colors in order to clarify them for specific operating room.

*Wrong packaging material*: Different packing materials were used for instrument package based on different needs of the operation room. For example, surgical instruments used occasionally are usually packed using double-layer non-woven cloth. Instruments used frequently are packed using double-layer cotton cloth, in consideration that packaging material could be used to prepare sterile operation bed. CSSD staff might misuse the packing material if they do not follow strictly the introduction in the instrument list. *Indicator card missing*: Chemical indicator card is not placed inside the instrument package. *Wrong count of instrument*: The instrument packages are assembled with specific amount of instruments according to each surgical requirement.

When staff pack instruments, mistakes might occur if they mix up the required quantities of different instruments. For example, when a package contains 4 pieces of 14 cm kelly clamp and 2 pieces of 16 cm kelly clamp, staff might mistakenly allocate 2 pieces of 14 cm kelly clamp and 4 pieces of 16 cm kelly clamp, although the total number of instruments was correct.

### Surgical instrument tracking systems

The system comprises the quality monitoring model for instrument packaging, which records the detailed information including packaging time, hospital staff in charge, name of wrong instrument, count of wrong instrument, and so on. Packaging errors data are regularly recorded by an audit working group in the department, who perform a weekly audit control on 50 packages. Another source of packing errors comes from complaints by the operation room.

Tracking systems can also allow hospital personnel to track the status of surgical devices, make optimal use of instrument sets, expedite instrument turnaround, and access manufacturers’ instructions. The package tag is confirmed firstly while a packaging error is found or reported. In the surgical instrument tracking system, under the “tracking management” model, all detailed information regarding this packaging can be found under the item “tracking and status of disinfection package”.

### Statistical analysis

The distribution of packaging errors was shown as frequency and percentage. Packaging errors were also stratified by different hierarchies of nurses, time periods (8:00–12:00, 12:00–16:00, and 16:00–20:00), and clinical departments (Orthopedics, Gynecology and Obstetrics, General Surgery, Neurosurgery, Thoracic Surgery, Cardiovascular Surgery, and Urinary Surgery). In our hospital, nurses are graded into four levels based on working years: N1(< 1 year), N2 (1–5 years), N3 (≥5 years), and N4 (≥5 years; N4 level nurses are responsible for management and quality control, and are not involved in the packing process). Chi-squared test was applied to compare the differences of packing error rate between various stratifications. All analyses were conducted using the SPSS version 19.0 statistical package (SPSS Inc., Chicago, IL, USA).

## Results

A total of 398 (1.18%) packaging errors were identified out of 33,839 packages recorded in the surgical tracking system during January–August 2016 in our hospital.

Detailed reasons for packaging errors are listed in Table 1, and include package incomplete (*n* = 70;17.6%), instrument missing (*n* = 77;19.4%), instrument malfunction (*n* = 27;6.8%), instrument in wrong specification (*n* = 175;44.0%), wrong packaging tag (*n* = 8;2.0%), box and cover mismatched (*n* = 14;3.5%), wrong packaging material (*n* = 15;3.8%), indicator card missing (*n* = 6;1.5%), and wrong count of instrument (*n* = 6;1.5%).

**Table 1:** Type of packaging errors during surgical instrument processing

Reasons	n	%
Package incomplete	70	17.6%
Instrument missing	77	19.4%
Instrument malfunction	27	6.8%
Instrument in wrong specification	175	44.0%
Wrong packaging tag	8	2.0%
Box and cover mismatched	14	3.5%
Wrong packaging material	15	3.8%
Indicator card missing	6	1.5%
Wrong count of instrument	6	1.5%

N1, N2, and N3 level nurses were responsible for 13,512 (39.9%), 11,425 (33.8%), and 8902 (26.3%) instrument packages, respectively (Table 2). Packing errors by N1 level nurses accounted for 69.35% of all errors found. Meanwhile, the error rate (276/13,512; 2.04%) was also highest in N1 level nurses compared to N2 (87/11,425; 0.76%) and N3 (35/8902; 0.39%) level nurses (*p* < 0.001).

**Table 2:** Surgical instrument packaging errors by different hierarchies of nurses

Hierarchy	Total package (%)	Package with errors (%)	Error rate
N1	13,512 (39.9%)	276 (69.4%)	2.04%*
N2	11,425 (33.8%)	87 (21.8%)	0.76%
N3	8902 (26.3%)	35 (8.8%)	0.39%

*p < 0.05

The volume of surgical instruments packed was nearly equal during the 8:00–12:00 (29.6%), 12:00–16:00 (32.7%), and 16:00–20:00 (37.7%) time periods (Table 3). During the 16:00–20:00 time period, a total of 172 packages with errors were found, accounting for 43.2% of the total errors found. The error rate was 1.35% (172/12,757) during this time period, and higher than both of the other time periods (*p* = 0.005).

**Table 3:** Surgical instrument packaging errors by different time periods

Time period	Total package	Package with errors	Error rate
8:00–12:00	10,025 (29.6%)	89 (22.4%)	0.89%
12:00–16:00	11,057 (32.7%)	137 (34.4%)	1.24%
16:00–20:00	12,757 (37.7%)	172 (43.2%)	1.35%*

*p < 0.05

**Fig. 1:**
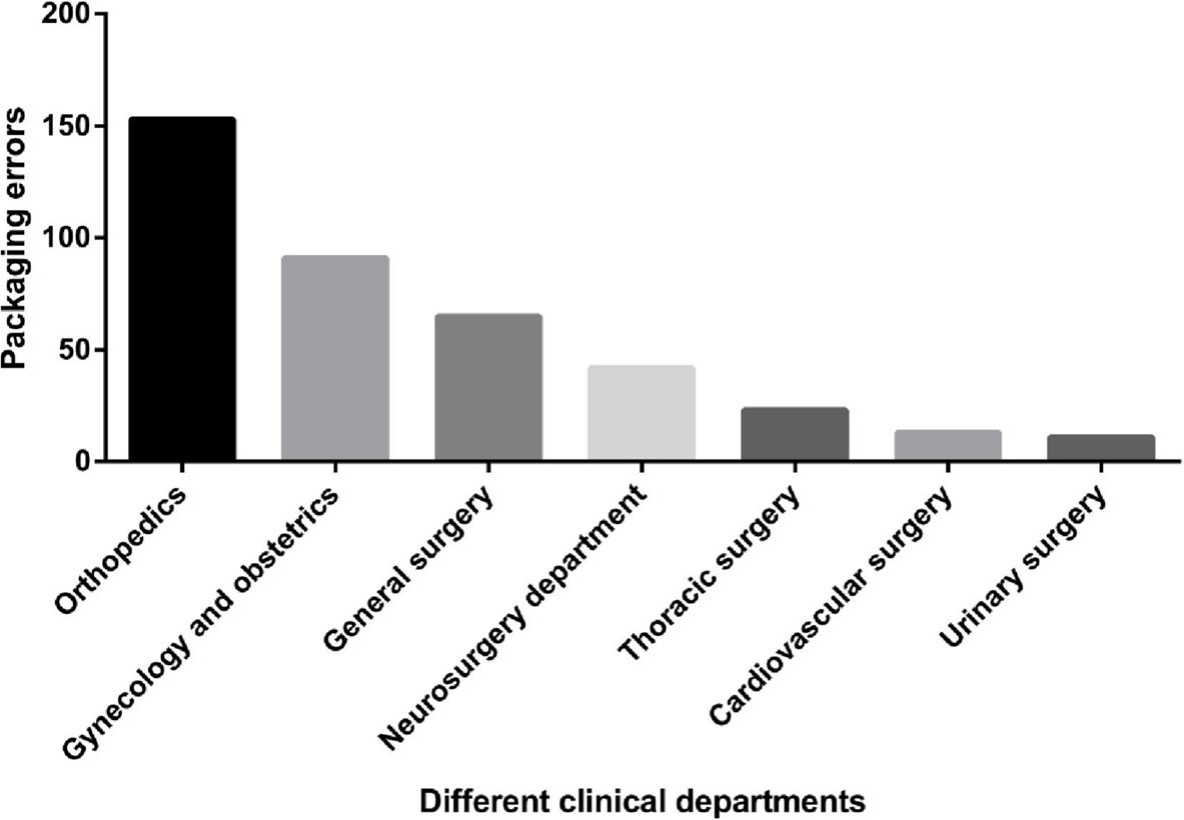
Surgical instrument packages with errors in different clinical departments

Departments of Orthopedics used 8472 packages (25.0%) and General Surgery used 10,014 packages (29.5%), followed by Department of Gynecology and Obstetrics (19.0%) (Table 4). Interestingly, although the Department of General Surgery used the most instrument packages, its error rate was among the lowest (65/10,014; 0.65%), whereas the Department of Orthopedics had the highest error rate (1.81%). Department of Urinary Surgery only used 1020 (3.0%) packages, however a high error rate (11/1020; 1.08%) was observed with them. The distribution of instrument package with errors is illustrated in Fig. 1.

**Table 4:** Surgical instrument packaging errors by different clinical departments

Departments	Total package	Package with errors	Error rate
Orthopedics	8472 (25.0%)	153 (38.4%)	1.81%
Gynecology and obstetrics	6429 (19.0%)	91 (22.9%)	1.42%
General surgery	10,014 (29.5%)	65 (16.3%)	0.65%
Neurosurgery department	3465 (10.2%)	42 (10.6%)	1.21%
Thoracic surgery	2402 (7.1%)	23 (5.8%)	0.96%
Cardiovascular surgery	2037 (6.0%)	13 (3.3%)	0.64%
Urinary surgery	1020 (3.0%)	11 (2.8%)	1.08%

## Discussion

Accurate management of surgical instruments packages plays a key role in decreasing the risk of operating room miscounting and malfunctions. Indeed, packaging errors can not only interrupt the operation, but also increase the cost.

In our hospital, 44.0% of errors occurred due to the wrong instrument specification packed, which mainly happened among the same type of instruments. The same type of instruments are frequently mixed due to their similar structure. The differences in structure of these instruments are so minute that they are hardly distinguishable. For example, the differences of various types of “nerve hook stripping ion” can be as small as 3 mm. Furthermore, the design of such a surgical instrument is so exquisite that the tag attached by manufacturers has a very small font, which can easily be misread after frequent cleaning and sterilization.

Detachable parts of surgical instruments are cleaned separately, and assembled before the packaging [15]. Errors easily occur when the same type of instruments are assembled. For example, the package for neurosurgery aneurysm operation contains two pieces of aneurysm clamp, which are easily mixed when they are assembled. Although the two pieces of aneurysm clamp are in different sizes, they are much alike in the inner core with minute differences, thus raising the difficulty in assembling them.

Not surprisingly, N1 level nurses, who have the least working experience, had a statistically significant higher error rate than other more advanced level nurses. Interesting, we also noticed that the highest error rate was found during the 16:00–20:00 time slot, when mostly N1 level nurses work. When analyzed by different clinical departments, the highest error rate was detected in the Orthopedics Department, which can be explained mainly by the fact that larger quantities of same category instruments were used in this department. Additionally, a large proportion of surgical instruments in this department were rented, and an indicator card was required for these rented instrument packages, which also increased the risk of error.

Personnel error is the primary reason for packaging errors. CSSD staff members are not familiar with the clinical utilization of surgical instruments, and therefore it was hard for them to distinguish between instruments with minor differences. In addition, relatively loud noise levels, ranging from 85 dB to 95 dB, were identified in the CSSD. This intensity is higher than the maximal cutoff value defined by the Law of the People’s Republic of China on the Prevention and Control of Ambient Noise Pollution. Long working under strong noise pollution can induce fatigue, dysphoria, and hearing impairment, which to some extent increases the risk of error [16]. High workload was also suggested as a potential reason leading to personnel error. Before July 2010, only four staff took charge of surgical instrument management, while the nurse on duty needed to additionally manage the counting and packaging of instruments by seeking assistance from an outside company [17]. Work overload involving hospital staff focusing on counting a large number of instruments, (and therefore missing and wrongly counting instruments) comprises a large proportion of the packaging errors.

Surgical instrument packaging error is a barrier to the high quality and safety of surgical care, and therefore efforts shall be addressed to prevent these amendable errors. Indeed, reported 30 to 50% of packaging errors could be prevented according to research by Jingmiao and Ping (2010) [18]. Recently it has been reported that Lean methodology could improve the quality of surgical instrument processing with a combination of various management parts, including redefining operator roles, alteration of the workplace, mistake-proofing, quality monitoring, staff training, and continuous feedback [3]. By using Lean production improvement methods, called the Virginia Mason Production System (VMPS), instrument processing errors in one study decreased from 3.0 to 1.5%, particularly the assembly errors of packaging (from 0.66 to 0.24 errors per 100 cases) [3] Furthermore, a training program could be initiated to interpret the differences for those instruments under the same category, and their specific application in clinics, in order to improve the familiarity and understanding of instrument for hospital personnel [19]. Recently, a training program designed for specific group of packaging staff has been shown to be helpful to improve their instrument knowledge [20].

The packaging error rate observed in the current study (1.18%) was similar to that observed in the United States (1.5%) [3]. These results indicated that hundreds of thousands of error had occurred during surgical procedures each year in China, United States, and other countries. Although there is lack of evidence proving that these errors had influenced patient’s outcome directly, the operative (and anesthesia) time could be increased due to these errors, which might increase the risk of complications and wound contamination. Therefore, identification and correction of these errors are important for improving surgical quality and safety.

## Conclusion

In summary, the current study identified that instruments in the wrong specifications, incomplete packaging, and instruments missing from the packages are the most common packaging errors in our hospital. N1 level nurses had a statistically significant higher error rate than the more experienced N2 and N3 level nurses. Further effort is needed to improve packaging procedure monitoring for instruments under the same category.

## Abbreviations

CSSD: Central sterile supply department
RFID: Radio-frequency identification
VMPS: Virginia Mason Production System
